# Whole Genome Sequences of Nine *Xanthomonas* Strains Responsible for Common Bacterial Blight of Bean

**DOI:** 10.1128/mra.01259-22

**Published:** 2023-02-13

**Authors:** Charlotte Gaudin, Christopher Gihaut, Martial Briand, Abi S. A. Marques, Marisa A. S. V. Ferreira, Marie-Agnès Jacques, Nicolas W. G. Chen

**Affiliations:** a Université Angers, Institut Agro, INRAE, IRHS, SFR QUASAV, Angers, France; b Plant Quarantine Station, Embrapa Recursos Genéticos e Biotecnologia, Brasília, Brazil; c Departamento de Fitopatologia, Universidade de Brasília, Brasília, Brazil; University of Arizona

## Abstract

We report the complete and circularized genome sequences of nine strains of Xanthomonas phaseoli pv. phaseoli and Xanthomonas citri pv. fuscans, which cause common bacterial blight of bean. These assemblies provide high-quality material for functional and evolutionary studies of these legume pathogens.

## ANNOUNCEMENT

Common bean (Phaseolus vulgaris) is an important legume crop for direct human consumption that is used worldwide for its edible pods and seeds (1). Common bacterial blight (CBB) is a devastating seed-borne bacterial disease that represents an important economic and agricultural threat to both common bean production and the seed industry worldwide (2). CBB is caused by two phylogenetically distant bacterial species that cause identical symptoms on common bean, i.e., Xanthomonas phaseoli pv. phaseoli and Xanthomonas citri pv. fuscans (2). These two species are subdivided into four genetic lineages. X. phaseoli pv. phaseoli corresponds to lineage GL1, while X. citri pv. fuscans comprises lineages GL2, GL3, and fuscans (2, 3).

Here, we produced Pacific Biosciences (PacBio) high-fidelity (HiFi) genome sequences of nine strains that had been isolated from Phaseolus vulgaris plants in different regions of the world and belonged to either GL1, GL2, or fuscans lineages (Table 1). Strains were isolated from symptomatic leaflets as described previously (4). The status as a CBB agent was confirmed by pathogenicity tests and sequencing of the housekeeping gene *gyrB* (5). Bacteria were grown on Trypticase soy (TS) agar medium for 2 days at 28°C, and then 5 or 6 clones were scraped, pooled, and cultured overnight in 100% TS broth to obtain fresh cultures. One milliliter of overnight culture was centrifuged for 2 min at 13,000 × *g*. Genomic DNA was extracted using the Wizard genomic DNA purification kit (Promega) according to the manufacturer’s recommendations. DNA was mechanically sheared using g-TUBE columns (Covaris) and purified using PacBio AMPure PB beads. PacBio HiFi SMRTbell libraries were prepared from ~1 μg of genomic DNA using the SMRTbell Express template preparation kit v2.0. Single-molecule real-time (SMRT) sequencing was performed on a PacBio Sequel II system using the Sequel II binding kit v2.2, sequencing primer v5, and Sequel II sequencing plate v2.0.

**TABLE 1:** Accession numbers and statistics

Pathovar	Lineage	Strain	Country (date)of collection	No. ofreads^*a*^	Coverage(×)	*N*_50_(bp)^*a*^	SRAaccession no.	GenBankassembly no.	Size(bp)	G+Ccontent (%)	No. ofplasmids	No. ofCDSs	Source orcollection
X. phaseoli pv. phaseoli	GL1	UnB187	Brazil (1981)	40,024	61	10,755	SRR22094557	GCA_025987765.1	5,217,655	64.9	2	4,187	This study
X. phaseoli pv. phaseoli	GL1	BB007a	USA (2007)	94,609	141	10,404	SRR22094556	GCA_025987745.1	5,242,207	64.8	1	4,236	This study
X. phaseoli pv. phaseoli	GL1	CFBP 7423	Iran (2006)	73,335	111	10,510	SRR22094555	GCA_025987785.1	5,214,828	64.8	3	4,209	CIRM-CFBP^*b*^
X. phaseoli pv. phaseoli	GL1	BB075a	USA (2014)	86,101	130	10,373	SRR22094554	GCA_025987725.1	5,161,803	64.9	2	4,145	This study
X. phaseoli pv. phaseoli	GL1	BB013a	USA (2007)	118,064	177	10,200	SRR22094553	GCA_025987685.1	5,199,960	64.8	2	4,194	This study
X. citri pv. fuscans	GL2	CFBP 6533	Tunisia (1989)	71,377	103	10,250	SRR22094552	GCA_025987705.1	5,154,616	64.6	1	4,209	CIRM-CFBP^*b*^
X. citri pv. fuscans	GL2	BRM48906	Brazil (2014)	42,986	64	10,574	SRR22094551	GCA_025987665.1	5,230,237	64.6	2	4,234	This study
X. citri pv. fuscans	GL2	LNPV 30.30	China (2006)	93,288	146	10,736	SRR22094550	GCA_025987645.1	5,156,952	64.6	1	4,212	This study
X. citri pv. fuscans	Fuscans	X1008017	France (2010)	144,222	211	10,204	SRR22094549	GCA_025987485.1	5,101,251	64.7	2	4,038	This study

a Calculated with the n50 tool (https://github.com/quadram-institute-bioscience/seqfu/wiki/n50).

b CIRM-CFBP, International Centre for Microbial Resources-French Collection for Plant-Associated Bacteria, INRAE (https://doi.org/10.15454/E8XX-4Z18).

PacBio reads were filtered and demultiplexed using the ccs v6.3.0 and lima v2.5.1 tools of the PacBio SMRT Tools v11.0.0.146107 toolkit and then assembled and circularized using Flye v2.9 (https://github.com/fenderglass/Flye) (6). For the BB007a strain, assembly and circularization were performed again using preselection of reads with Filtlong (https://github.com/rrwick/Filtlong). Circularization failed for strains BRM48906 and UnB187. For circularized genomes, the sequence start was fixed using the fixstart option of Circlator v1.5.1 (7). Polishing was performed using Flye v2.9 (6).

The genome sequences consisted of 5,101,251 to 5,242,207 bp, forming a chromosome plus 1 to 3 plasmids, with an average G+C content of 64.7% (Table 1). A total of 4,038 to 4,236 predicted protein-coding sequences (CDSs) were identified using the NCBI Prokaryotic Genome Annotation Pipeline (PGAP) (8). Genome sequences were estimated to be >99.3% complete and <0.02% contaminated using CheckM v1.1.6 (9). Chromosomes were assembled in 1 contig except for strain X1008017, with 2 contigs. Every strain contained plasmid A, which is consistent with previous data for CBB agents (10) and reinforced the idea that plasmid A was involved in the pathological convergence on bean by these two species (11). Each genome sequence included the usual arsenal of genes involved in pathogenicity, compared to the reference strain CFBP 4834-R (12), although the GL1 and GL2 lineages showed polymorphism in important components such as the lipopolysaccharide and secretion systems. These assemblies provide high-quality material for functional and evolutionary studies of these common bean pathogens.

### Data availability.

The reads and assemblies were all deposited in GenBank under the accession numbers listed in Table 1.

## References

[1] Bitocchi E, Rau D, Bellucci E, Rodriguez M, Murgia ML, Gioia T, Santo D, Nanni L, Attene G, Papa R. 2017. Beans (*Phaseolus* ssp.) as a model for understanding crop evolution. Front Plant Sci 8:722. doi:10.3389/fpls.2017.00722. 28533789 PMC5420584

[2] Chen NWG, Ruh M, Darrasse A, Foucher J, Briand M, Costa J, Studholme DJ, Jacques MA. 2021. Common bacterial blight of bean: a model of seed transmission and pathological convergence. Mol Plant Pathol 22:1464–1480. doi:10.1111/mpp.13067. 33942466 PMC8578827

[3] Alavi SM, Sanjari S, Durand F, Brin C, Manceau C, Poussier S. 2008. Assessment of the genetic diversity of *Xanthomonas axonopodis* pv. phaseoli and *Xanthomonas fuscans* subsp. *fuscans* as a basis to identify putative pathogenicity genes and a type III secretion system of the SPI-1 family by multiple suppression subtractive hybridizations. Appl Environ Microbiol 74:3295–3301. doi:10.1128/AEM.02507-07. 18359831 PMC2394934

[4] Jacques M, Josi K, Darrasse A, Samson R. 2005. *Xanthomonas axonopodis* pv. phaseoli var. fuscans is aggregated in stable biofilm population sizes in the phyllosphere of field-grown beans. Appl Environ Microbiol 71:2008–2015. doi:10.1128/AEM.71.4.2008-2015.2005. 15812033 PMC1082538

[5] Young JM, Park DC, Shearman HM, Fargier E. 2008. A multilocus sequence analysis of the genus *Xanthomonas*. Syst Appl Microbiol 31:366–377. doi:10.1016/j.syapm.2008.06.004. 18783906

[6] Kolmogorov M, Yuan J, Lin Y, Pevzner PA. 2019. Assembly of long, error-prone reads using repeat graphs. Nat Biotechnol 37:540–546. doi:10.1038/s41587-019-0072-8. 30936562

[7] Hunt M, De Silva N, Otto TD, Parkhill J, Keane JA, Harris SR. 2015. Circlator: automated circularization of genome assemblies using long sequencing reads. Genome Biol 16:294. doi:10.1186/s13059-015-0849-0. 26714481 PMC4699355

[8] Tatusova T, Dicuccio M, Badretdin A, Chetvernin V, Nawrocki EP, Zaslavsky L, Lomsadze A, Pruitt KD, Borodovsky M, Ostell J. 2016. NCBI Prokaryotic Genome Annotation Pipeline. Nucleic Acids Res 44:6614–6624. doi:10.1093/nar/gkw569. 27342282 PMC5001611

[9] Parks DH, Imelfort M, Skennerton CT, Hugenholtz P, Tyson GW. 2015. CheckM : assessing the quality of microbial genomes recovered from isolates, single cells, and metagenomes. Genome Res 25:1043–1055. doi:10.1101/gr.186072.114. 25977477 PMC4484387

[10] Ruh M, Briand M, Bonneau S, Jacques MA, Chen NWG. 2017. *Xanthomonas* adaptation to common bean is associated with horizontal transfers of genes encoding TAL effectors. BMC Genomics 18:670. doi:10.1186/s12864-017-4087-6. 28854875 PMC5577687

[11] Chen NWG, Serres-Giardi L, Ruh M, Briand M, Bonneau S, Darrasse A, Barbe V, Gagnevin L, Koebnik R, Jacques MA. 2018. Horizontal gene transfer plays a major role in the pathological convergence of *Xanthomonas* lineages on common bean. BMC Genomics 19:606. doi:10.1186/s12864-018-4975-4. 30103675 PMC6090828

[12] Darrasse A, Carrère S, Barbe V, Boureau T, Arrieta-Ortiz ML, Bonneau S, Briand M, Brin C, Cociancich S, Durand K, Fouteau S, Gagnevin L, Guérin F, Guy E, Indiana A, Koebnik R, Lauber E, Munoz A, Noël LD, Pieretti I, Poussier S, Pruvost O, Robène-Soustrade I, Rott P, Royer M, Serres-Giardi L, Szurek B, van Sluys MA, Verdier V, Vernière C, Arlat M, Manceau C, Jacques MA. 2013. Genome sequence of *Xanthomonas fuscans* subsp. *fuscans* strain 4834-R reveals that flagellar motility is not a general feature of xanthomonads. BMC Genomics 14:761. doi:10.1186/1471-2164-14-761. 24195767 PMC3826837

